# Sustainable Practices in Anti-VEGF Therapy: A 15-Year Bibliometric Analysis of Ranibizumab for Age-Related Macular Degeneration

**DOI:** 10.1155/joph/8891531

**Published:** 2025-05-06

**Authors:** Li Xiaodong, Chen Xia, Qin Xuewei, Wu Dandan, Yang Yi, Li Zhilin

**Affiliations:** ^1^Department of Ophthalmology, The First Affiliated Hospital of Guizhou University of Traditional Chinese Medicine, Guiyang, China; ^2^Department of Nephrology, West China Hospital, Sichuan University, Chengdu, China; ^3^West China School of Nursing, Sichuan University, Chengdu, China; ^4^Department of Ophthalmology, Chengdu University of Traditional Chinese Medicine, Chengdu, China

**Keywords:** aflibercept, age-related macular degeneration, bevacizumab, endothelial growth-factor, ranibizumab, sustainable practices

## Abstract

**Objective:** A bibliometric analysis was performed in the domain of ranibizumab and age-related macular degeneration (AMD) to delineate current trends in international research dynamics and to provide a visual representation of research hotspots and challenges associated with ophthalmic drugs over the past 15 years. This study also evaluates the sustainability of ranibizumab therapy through reduced injection burden, cost-effectiveness compared to alternative treatments, and long-term outcomes that minimize healthcare resource utilization.

**Method:** In this cross-sectional study, bibliometrics analyzed data retrieved and extracted from the Web of Science Core Collection (WOSCC) database to analyze the evolution and thematic trends in the delivery of studies from January 1, 2008, to September 2, 2023, for ranibizumab and AMD studies. A total of 2691 articles on the field were assessed for specific characteristics such as the year of publication, journal, author, institution, country/region, citation, and keywords. Co-authorship analysis, co-occurrence analysis, co-citation analysis, and network visualization were constructed using VOSviewer. Some important subtopics identified by bibliometric characterization were further discussed and reviewed.

**Results:** From 2008 to 2023, the cumulative number of articles published globally increased from 1 to 2,691, with the highest number of articles published in 2020 (255 papers). RETINA THE JOURNAL OF RETINAL AND VITREOUS DISEASES published the most manuscripts (285 papers) and was cited (6496 citations), followed by OPHTHALMOLOGY (193 papers) and GRAEFES ARCHIVE FOR CLINICAL AND EXPERIMENTAL OPHTHALMOLOGY (163 papers). OPHTHALMOLOGY was the most cited (20,865 citations), with the United States (786 papers, 38,014 citations), Univ Sydney (98 papers, 5245 citations), and Kim, Jong Woo (56 papers, 550 citations) being the most productive and influential institutions, countries, and authors, respectively. Five clusters were formed by summarizing the top 100 keywords, which marked the emerging frontier of ranibizumab and AMD-related research. Further discussion of the five clusters of research is to assist the researcher in determining the scope of the research topic and planning the direction of the research.

**Conclusion:** Over the past two decades, there has been a notable increase in the number of publications and citations pertaining to ranibizumab and AMD across various countries, institutions, and authors. This study elucidates current trends, global collaboration patterns, foundational knowledge, research hotspots, and developmental trajectories within the realm of ranibizumab-related AMD research. Key advancements in AMD treatment with ranibizumab over the last 15 years have centered on less frequent injection schedules, extended drug efficacy, and enhanced safety profiles.

## 1. Introduction

AMD is one of the common refractory fundus diseases resulting in irreversible vision loss and even blindness in the elderly, which involves the pigment epithelium, photoreceptor cell layer, and choroidal multilayers in the macular area of the retina. The prevalence of AMD is increasing with the intensification of the global population aging [1]. AMD is a complex, blinding disease induced by multiple factors, and it has been shown that the pathogenesis of AMD involves oxidative stress, endoplasmic reticulum stress, impaired autophagy, mitochondrial dysfunction, and inflammation [2, 3]. Currently, AMD is classified into dry AMD and wet AMD according to its pathology. Wet AMD, also known as neovascular age-related macular degeneration (nAMD), was characterized by the main pathological feature of macular choroidal neovascularization (MNV). MNV can exacerbate the damage of the center of the visual acuity and result in a severe loss of visual acuity [4]. Vascular endothelial growth factor (VEGF) is an important inflammatory factor that induces the development of MNV, and vitreous cavity injection of anti-VEGF drugs is the first-line treatment option for nAMD in various guidelines and expert consensus from the molecular biological mechanism of nAMD pathogenesis [5]. Ranibizumab is currently the most widely used drug with better evidence-based medicine. Vitreous injection of ranibizumab can inhibit VEGF and block MNV growth and leakage, which has shown a definite efficacy in the treatment of AMD [6]. Sustainability in ophthalmology encompasses not only environmental considerations but also the optimization of therapeutic regimens to reduce patient burden, improve cost-efficiency, and ensure equitable access to care. The shift toward less frequent dosing schedules and personalized treatment plans for AMD reflects a growing emphasis on sustainable clinical practices.

Although there have been multiple reviews on ranibizumab and AMD with different emphases, a comprehensive and visualized analysis of the evolution and trends of ranibizumab and AMD is still lacking. Bibliometric analysis is a scientific, quantitative method of researching publications, including co-word analysis, social network analysis, and cluster analysis, which summarizes the progress on a research topic and detects hot or emerging trends and contributions from authors, journal institutions, or countries using quantitative statistics [7]. Utilizing bibliometric analysis, we first characterized the current status of research fields in ranibizumab and AMD and explored the trend. We aimed to identify the current hotspots in this area to offer a perspective into future research topics. Publications are obtained from the Web of Science Core Collection (WOSCC) from 1985 to 2023 in terms of the distribution of annual publications, countries, institutions, authors, source journals, keyword co-occurrence, and co-citation. Furthermore, we provided further discussions into some important subtopics identified by bibliometric characterization. This study would assist both new researchers and specialists to determine the range of research topics, identify new topics, or plan research direction in the field of ranibizumab and AMD.

## 2. Materials and Methods

**Figure 1:**
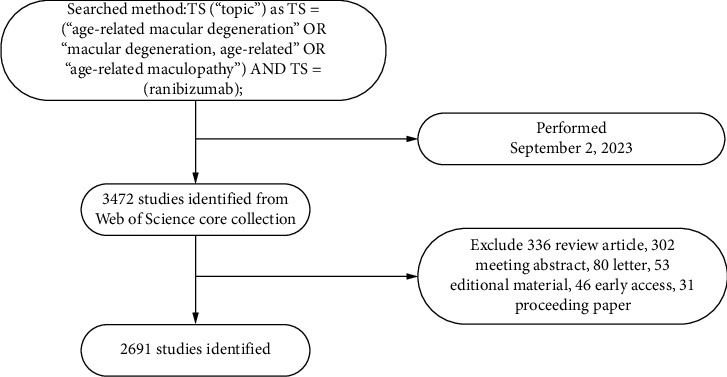
The data collection and retrieval strategy.

The main search method and process of literature included in this study, data retrieval for all included literature was completed within 1 day on September 2, 2023 and downloaded as “text” from WoSCC [8]. Although other databases, such as PubMed and Scopus, are also widely used in the biomedical field, WOSCC was chosen for this study mainly because of its advantages in multidisciplinary high-quality literature collection and its unified indexing system, which facilitates the accurate retrieval of related literature and ensures the consistency of the data sources. However, there are some limitations; for example, it may miss some documents that are only included in other databases. A cross-database comparative analysis can be considered in subsequent studies to further validate the results of the study.  Figure 1 shows the main process of literature search and data extraction in this study.

Inclusion criteria:

1. The literature subject line search formula is as follows: (“Age-Related Macular Degeneration” OR “Macular Degeneration, Age-Related” OR “Age-Related Maculopathy”) AND (Ranibizumab);
2. The type of study included was limited to “article”;
3. Inclusion of research publication years limited to 1985–2023;
4. Key information was extracted from the included studies, including publication, first and corresponding authors, countries/region, research institutions, journals, and keywords.

During data retrieval, only one search strategy was used and different search algorithms were not tested. A follow-up study is planned to explore multiple search strategies, such as adjusting search terms, changing Boolean operator combinations, etc., and compare the search results under different strategies to verify the stability of the search results.

### 2.1. Data Analysis and Network Mapping

In this study, VOSviewer version 1.6.19 was used to extract information such as researcher, research institution, country/region, citations, and keywords from TXT files downloaded from the WoSCC website to generate a visualized network diagram [9]. Commonly used bibliometric techniques include co-authorship analysis, citation co-occurrence analysis, and keyword co-occurrence analysis to identify themes and research hotspots in the literature. Co-authorship analysis reveals the pattern and depth of collaboration between authors, institutions, and countries/regions [10]. Co-occurrence analysis uses the frequency of multiple keywords in the same article to determine the extent to which they are connected, thus showing hot topics and trends in the discipline. For topics of interest, these visualizations enable researchers to identify active authors, institutions, countries, foundational knowledge, research hotspots, research frontiers, and other bibliometric information [11]. In the VOSviewer visualization map, each node is represented by a circle with a label. In the co-occurrence analysis, larger circles indicate higher frequency. The color of each circle is determined by the cluster they belong to. The thickness and length of inter-node links represent the connection strength and correlation between the corresponding nodes [8].

### 2.2. Research Ethics

The literature data included in this study are publicly available from the WOSCC, and the acquisition of information from these data did not involve interaction with human subjects or animals. Therefore, this study did not require ethics committee approval [8].

## 3. Results

### 3.1. Annual Global Publication Outputs on Ranibizumab and AMD

**Figure 2:**
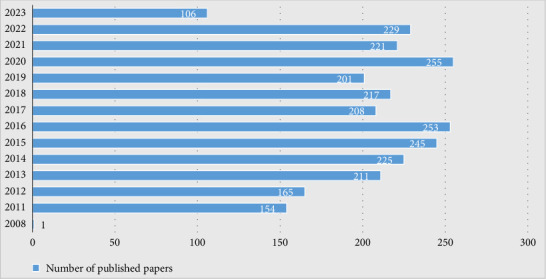
The number of articles on ranibizumab and AMD per year from 2008 to 2023.

As of September 2, 2023, a total of 2691 ranibizumab and AMD-related treatises were retrieved from the WOC database, and the number of publications per year is shown in Figure 2. There was only one article in 2008, and no publication was seen in the following 2 years. Then, the number of publications soared to 154 articles in 2011, followed by a gradual upward trend. Scholars' interest in the research topic of ranibizumab and AMD. The number of publications was 253 in 2016, although the number of publications gradually decreased from 2017 to 2019, but it was stable at more than 200. And the number of publications peaked at 255 in 2020, which may be related to the global outbreak of the novel coronavirus epidemic, so the researchers have more sufficient time to write and publish papers. The number of publications in 2021 and 2022 slightly decreased to 221 and 229. As of now, 106 have been published in 2023, indicating that ranibizumab and AMD-related studies are the research hotspots and difficulties at present. The peak publication year (2020) coincided with global efforts to reduce healthcare costs during the COVID-19 pandemic, highlighting the role of sustainable treatment strategies like extended dosing intervals in crisis resilience.

### 3.2. Distribution of Source Journals and Top 10 High-Cited Articles

The retrieved articles on ranibizumab and AMD were published in 306 journals.  Table 1 lists the top 10 journals in terms of the number of publications, accounting for 45.78% (1232/2691) of the total number of publications, RETINA THE JOURNAL OF RETINAL AND VITREOUS DISEASES is the journal with the highest number of publications (285), but the journal with the highest number of citations is OPHTHALMOLOGY (20,865), while it has the second highest number of publications (193).

The total number of citations for the 2691 articles included is 73,023, and the average number of citations per article is 27.13.  Table 2 lists the top 10 cited papers. The most cited paper is “Global prevalence of age-related macular degeneration and disease burden projection for 2020 and 2040: a systematic review and meta-analysis” (with 2561 citations), which was published in the journal LANCET GLOB HEALTH. Followed by “Ranibizumab and Bevacizumab for Neovascular Age-Related Macular Degeneration The CATT Research Group” (cited 2010), was published in the top international journal NEW ENGLAND JOURNAL OF MEDICINE.

In addition, calculating the average number of citations per year and the standard deviation of the number of citations provides a more complete assessment of the change in research impact over time. For example, the average number of citations for articles per year for the period 2011–2023 is 3154, and the standard deviation of citations is 556. The average number of citations reflects the average impact of research in the field across years, while the standard deviation reflects the degree of dispersion of citations, i.e., the stability of the impact of research.

### 3.3. Distribution and Co-Authorship of Countries/Regions

**Figure 3:**
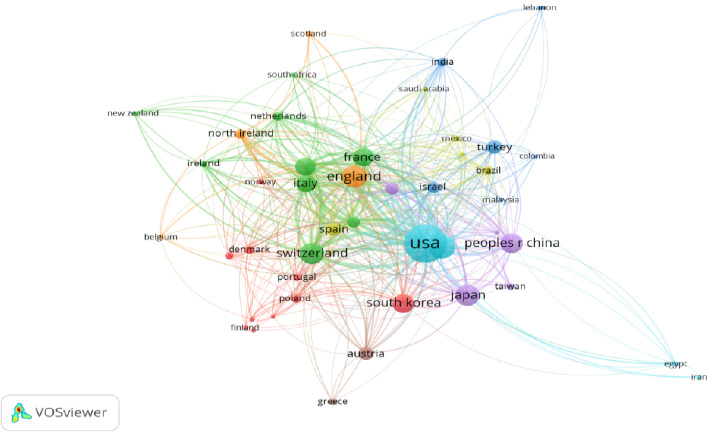
The coauthorship network of countries/regions. (Nodes of different colors in the graph may represent different cooperation clusters or research groups. Nodes of the same color may have a closer cooperation relationship with each other or have some similarity in research topics.).

Ranibizumab and AMD are a global research hot topic; currently, 72 countries are involved in this research; Figure 3 shows the distribution of multinational collaborations in this field; the United States is at the center of the research collaboration in this field.  Table 3 lists the top 10 countries in terms of the number of publications in this field, with the United States currently ranking first with 786 publications and 38,014 citations, followed by Germany (319 publications and 12,583 citations) and the United Kingdom (277 publications and 10,862 citations).

### 3.4. Distribution and Co-Authorship of Institutions

**Figure 4:**
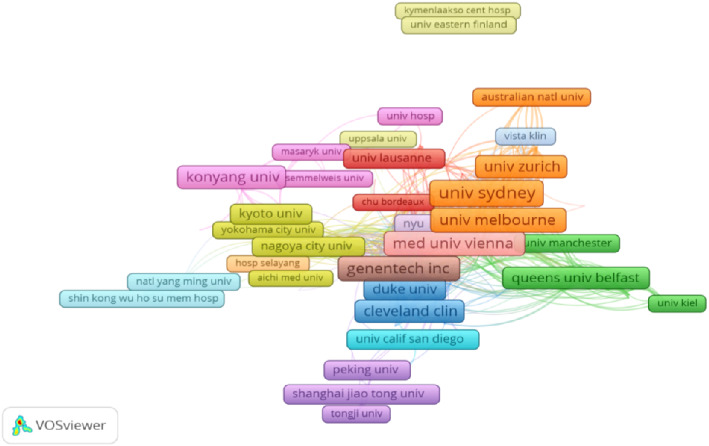
The coauthorship network of institutions. (Nodes of different colors in the graph may represent different cooperation clusters or research groups. Nodes of the same color may have a closer cooperation relationship with each other or have some similarity in research topics.).

A total of 3144 organizations were involved in the research related to ranibizumab and AMD, and Table 4 lists the top 10 organizations in the world with the highest number of publications in the field, with the University of Sydney, located in Australia, having the highest number of publications (98, cited 5245). A collaborative analysis of the 3144 organizations found that 370 of them were closely related to each other and were divided into 16 color-coded groups by VOSviewer, as shown in Figure 4.

### 3.5. Distribution and Co-Authorship of Authors

**Figure 5:**
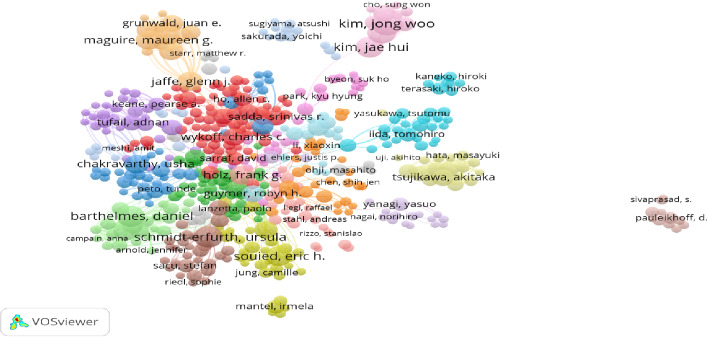
The coauthorship network of authors. (Nodes of different colors in the graph represent different collaborative clusters or research groups. Nodes of the same color often represent that these authors cooperate more closely with each other, or their research topics and directions are somewhat similar and belong to the same research circle.).

There are 10,197 authors in 2691 published articles, with an average of 4 authors per article.  Table 5 lists the top 10 authors with the most publications. Kim, Jong Woo is the first author with 56 publications, followed by Schmidt-Erfurth, Ursula (48) and Kim, Chul Gu (48), which are tied for second place, and Schmidt-Erfurth, Ursula has the highest number of citations for their published articles (5,890). As shown in Figure 5, the distribution of the relationships of the 503 authors who collaborate closely in this field was analyzed by the Vosviewer software, grouping them into 20 clusters with different colors.

### 3.6. The Co-Occurrence Analysis of the Top 10 and Top100 Keywords

**Figure 6:**
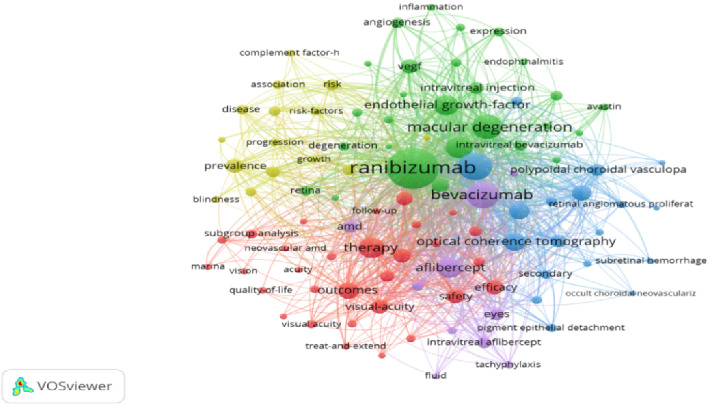
The co-occurrence cluster analysis of the top 100 keywords. (Nodes of different colors in the graph represent different keyword clusters. Nodes of the same color belong to the same cluster, meaning that these keywords are similar or related in terms of research topics or directions.).

**Figure 7:**
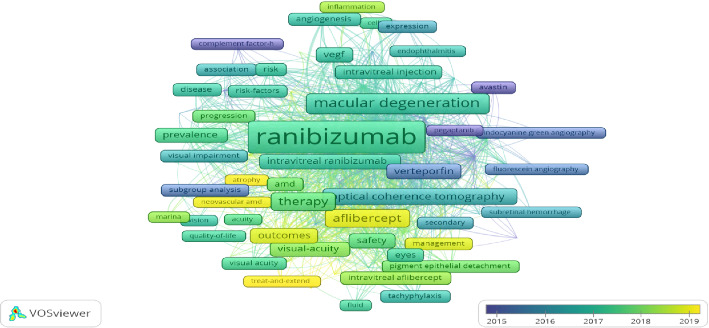
The overlay map of top 100 keywords. (Different color nodes constitute different clusters, and keywords of the same cluster are similar or related in terms of research topics or directions. The keywords' heat changes or research trends in different years.).

Keywords can best reflect the core theme of a paper, so it is appropriate to filter out the high-frequency keywords in 2691 articles to explore the research hotspots and research dynamics in this field.  Table 6 lists the top 10 high-frequency keywords in terms of the number of occurrences, with ranibizumab appearing the most frequently at 1919 times, followed by age-related macular regeneration at 831 times, and the third high-frequency keyword is bevacizumab at 812 times.  Figure 6 displays a visualization network map of the top 100 keywords in five clusters with their co-occurrence. The five clusters are shown in red (1), green (2), blue (3), yellow (4), and purple (5). Node labels indicate keywords, and the size of each node indicates the frequency of keyword occurrence. A link connecting two nodes indicates a co-occurrence relationship between two keywords. To explore the evolutionary trend over time, keywords retrieved from publications were colored according to their average occurrence year (AAY) using VOSviewer and coded using a heat map [8] (Figure 7). Cold-colored keywords represent early occurrences, while warm-colored keywords represent slightly later occurrences. The recent appeared keywords were treat and extend (AAY:2020.23), atrophy (AAY: 2019.05), morphology (AAY:2019.23), neovascular age-related macular degeneration (AAY: 2018.99), growth (AAY: 2018.94), neovascular AMD (AAY: 2018.84), aflibercept (AAY: 2018.77), 2.0 mg ranibizumab (AAY: 2018.73), macular (AAY: 2018.72), and management (AAY: 2018.66).

## 4. Discussion

### 4.1. Global Trends in Ranibizumab and AMD

From the analysis of the search results in this article, the research results related to ranibizumab and AMD were basically blank before 2011 and then suddenly entered a rapid development stage after 2011, and the number of articles published in this field was stable at about 200 per year from 2013 to 2022, which indicates that the research related to ranibizumab and AMD has been a hot research topic among scholars. Analyzing the quality of the top 10 journals in terms of publication volume, the top 9 journals are all high-quality international authoritative journals with JCR classification of Q1 and Q2, indicating that the research related to ranibizumab and AMD is indeed a key and difficult topic of interest for scholars in the field worldwide.

There are 10,197 authors from 3144 organizations and institutions in 72 countries around the world who have collaborated with each other to publish articles in this subject area. As shown in Figure 3, there is an extensive network of collaborative relationships among countries/regions, and the United States, as the country with the largest number of articles in this field, is at the core of international cooperation. In addition, analyzing clusters of different colors, the Asian countries with China, South Korea, and Japan as the core, and the European countries with the United Kingdom, Switzerland, France, and Italy as the core, all of which are at the forefront of the field. Among the top 10 most efficient institutions, there are four US institutions, two Australian institutions, two German institutions, and the remaining two institutions are from South Korea and Switzerland. The U.S. institutions are the major institutional organizations for the ranibizumab and AMD studies. As shown in Figure 4, the 370 institutions are labeled by different colors into 16 clusters, indicating that they are from 16 different countries/regions, e.g., orange clusters mainly represent institutions from Australia, and purple clusters represent institutions from China, and the complex network indicates that there are still extensive collaborations among these institutions from different countries. In addition, as shown in Table 5 and Figure 5, Kim, Jong Woo is the author with the most publications, however, but he is not the author with the highest number of citations or the highest number of collaborations, Jaffe, Glenn J is the author with the highest number of citations, and Barthelmes, Daniel is the author with the highest number of collaborations with others.

### 4.2. Hotspots and Emerging Frontiers on Ranibizumab and AMD

The co-occurrence analysis of keywords facilitates the categorization of major knowledge structures and hotspots in the current field. As shown in Figure 6 and Table 7, the co-occurrence clustering analysis of the top 100 keywords exhibits that there are five major research clusters for ranibizumab and AMD studies. In Figure 6 (co-occurrence cluster analysis of the top 100 keywords) and Figure 7 (overlay of the top 100 keywords), the different colored clusters represent the different areas of focus in the research on ranibizumab and AMD. The red clusters are mainly centered around the efficacy and safety of intravitreal injections of ranibizumab for the treatment of neovascular age-related macular degeneration, with the terms “treatment,” “outcome,” “safety,” “visual acuity,” and other keywords appeared with high frequency, reflecting the researchers' concern for determining the optimal injection dosage, dosing regimen, and evaluating the therapeutic efficacy. The green clustering focused on the mechanism of action of ranibizumab in the treatment of nAMD, and the keywords “vascular endothelial growth factor (VEGF),” “choroidal neovascularization (CNV),” and “anti-VEGF” indicated that this clustering mainly investigated the mechanism by which ranibizumab reduced VEGF levels and inhibited CNV growth. These clusters help researchers to clearly identify the main research directions in this field and the association between the research topics.

Cluster 1 (Figure 6, red cluster): The first study clustering contained 30 high-frequency keywords such as therapy, outcomes, neovascular age-related macula, safety, visual acuity, efficacy, intravitreal ranibizumab, trial, anti-vascular endothelial grow, etc. These keywords indicate that this research clustering mainly focuses on the efficacy and safety of intravitreal ranibizumab injection for neovascular age-related macula, selecting the appropriate dosage of ranibizumab injection, dosing regimen, and follow-up rules, improving patients' visual acuity and improving AMD patients' quality of life.

The efficacy of ranibizumab for the treatment of nAMD is certain, but there are two dosing regimens about ranibizumab for the treatment of nAMD, one is the 3 + PRN regimen, which refers to the three consecutive months of injections of one injection followed by a decision on whether to continue the injection treatment according to the condition, and the second refers to the treat-and-extend (T&E) regimen, which refers to the three consecutive months of injections; the second refers to the T&E program, which means that after one injection in three consecutive months, the interval between injections will be extended according to the condition. T&E is a process of active treatment between the doctor and the patient, with a high degree of benefit to the patient in the long run. Passive treatment (PRN), on the other hand, means that it refers to the on-demand treatment after completing the load treatment of three or five times, which is more risky for the patient. The T&E program effectively improves and maintains patients' vision over the long term, reduces recurrence, extends treatment intervals to a maximum of 16 weeks, reduces clinical complications due to injections, and lowers the burden of disease for patients; it is a better program for patients. A meta-analysis [22] of approximately 26,360 patients who reported intravitreal ranibizumab for nAMD outcomes in 42 real-world observational studies published between 2007 and 2015. The mean change in visual acuity during years 1, 2, and ≥ 3 for patients receiving the T&E regimen was +8.8 (95% CI: 5.8–11.8), +6.7 (95% CI: 3.2–10.1), and +5.4 (95% CI: −4.1–14.9), with T&E patients receiving more injections on average in the first year (6.9 vs. 4.7), but had fewer office visits (7.6 vs. 9.2). Baseline characteristics were similar for both dosing regimens. Of the 66,176 intravitreal injections, the incidence of endophthalmitis was reported in 17 cases (0.026%). The study demonstrated the safety of intravitreal injections of ranibizumab for the treatment of nAMD in preventing severe vision loss. Although patients may regain vision from baseline, the extent to which vision is maintained in the long term may depend on the frequency of injections. Therefore, the T&E regimen may be more suitable for patients with higher demands on vision and quality of life. A recent 12-month clinical study [23] found that 0.5 mg of ranibizumab given according to the T&E regimen was not statistically different in terms of improvement in visual acuity or safety compared to the monthly injectable dosing regimen, and that most of the improvement in BCVA in the treatment groups of the two dosing regimens occurred in the first 6 months and was maintained until the end of the study. The purpose of optimizing the treatment regimen of ranibizumab is to provide AMD patients with long-term and effective vision restoration and protection; to reduce the number of visits to the clinic, so as to reduce the burden on the patients, their families, and the hospitals; to make full and effective use of the limited healthcare resources; and to take into account the individual differences of the patients and formulate a personalized treatment plan.

Scholars have also conducted a series of studies on the optimal effective dose of ranibizumab for the treatment of nAMD, and the current commonly used dose for intravitreal injections of anti-VEGF drugs is 0.5 mg. A 24-month, multicenter, randomized, double-blind, active treatment-controlled, phase 3 trial [24] found no difference in clinical efficacy and safety between intravitreal injections of ranibizumab given 0.5 and 2.0 mg in the treatment of patients with nAMD and did not differ in clinical efficacy or safety in the treatment of patients with nAMD.

For nAMD, the T&E regimen is more advantageous than the 3 + PRN regimen in the treatment of ranibizumab, which can effectively improve and maintain patients' vision in the long term, reduce recurrence, maximize the interval between injections to 16 weeks, reduce clinical complications caused by injections, and alleviate the burden of disease on the patients, especially for patients who have high demands on vision and quality of life. Clinicians can prioritize the T&E regimen when developing a treatment plan.

Cluster 2 (Figure 6, green cluster): The second study clustering contained 28 high-frequency keywords such as ranibizumab, macular degeneration, choroidal neovascularization (CNV), VEGF, anti-VEGF, intravitreal bevacizumab, avastin, pegaptanib, etc. This section focuses on the mechanism by which the ranibizumab treatment of nAMD reduces VEGF levels and inhibits CNV. There are five members of the VEGF molecular family, namely, VEGF-A, VEGF-B, VEGF-C, VEGF-D, and placental growth factor (PLGF), among which VEGF-A is the most active and is closely related to CNV and exudative macular degeneration [25]. Ranibizumab is a second generation of recombinant mouse anti-VEGF antibody fragment with a relative molecular weight of 48 kD. It can bind to all VEGF-A subtypes and block their binding to VEGFR1 and VEGFR2 [26], thereby reducing vascular endothelial cell proliferation and vascular permeability, inhibiting neovascularization [27, 28].

Bevacizumab (trade name Avastin) is a humanized full-length monoclonal antibody against VEGF that binds to all VEGF isomers and has two binding sites with VEGF. In 2004, the US FDA approved the market for the treatment of colorectal tumors; its indications do not include AMD [29]; however, due to its relatively low price, it is widely used in the treatment of AMD “off-label.” A multicenter, single-blind, nonefficacy trial [13] was studied in which researchers randomly assigned 1208 patients with nAMD to receive intravitreal injections of ranibizumab or bevacizumab, either on a monthly schedule or assessed monthly as needed. Results showed that bevacizumab administered monthly was comparable to ranibizumab administered monthly, adding 8.0 and 8.5 letters, respectively. Bevacizumab on an as-needed basis was comparable to ranibizumab on an as-needed basis, with increases of 5.9 and 6.8 letters, respectively. The efficacy of on-demand ranibizumab was comparable to that of monthly bevacizumab. Mortality, myocardial infarction, and stroke rates were similar in patients treated with bevacizumab or ranibizumab. A higher proportion of patients in the bevacizumab group experienced serious systemic adverse events (primarily hospitalization) than in the ranibizumab group. In addition, a recent retrospective study [30] showed that alternating ranibizumab/bevacizumab injections four times every 2 weeks improved visual acuity and reduced macular thickness in some patients with refractory vascular AMD and PED. Bevacizumab has a higher molecular weight, and animal studies have shown that bevacizumab does not pass through the inner retinal border membrane after intravitreal injection. Pharmacokinetic studies showed that its plasma half-life was 8.68 d, and its residence time in the systemic blood was significantly longer than that of ranibizumab [31]. Theoretically, bevacizumab has a greater systemic risk, but multicenter, large-sample, randomized, large-scale clinical studies are needed.

Understanding that VEGF-A in the VEGF molecular family is closely related to CNV and exudative macular degeneration, ranibizumab, as a second-generation recombinant mouse anti-VEGF antibody fragment, is able to bind all VEGF-A isoforms, blocking their binding to VEGFR1 and VEGFR2 and thus inhibiting vascular endothelial cell proliferation, vascular permeability, and neovascularization. Although bevacizumab is a humanized full-length monoclonal antibody against VEGF that binds to all VEGF isoforms, it was originally used for the treatment of colorectal tumors, and its use in AMD is considered an “over-the-counter” drug. In terms of efficacy, multicenter studies have shown that monthly injections of bevacizumab are comparable to monthly injections of ranibizumab, that they are similar when injected on an as-needed basis, and that the efficacy of as-needed injections of ranibizumab is comparable to monthly injections of bevacizumab. However, the proportion of serious systemic adverse events (mainly hospitalizations) was higher in the bevacizumab group, and the systemic risk was theoretically greater due to its high molecular weight and long plasma half-life (8.68 d), which resulted in a significantly longer residence time in the systemic blood than that of ranibizumab. Clinicians need to consider factors such as drug efficacy, safety, and the patient's economic status when choosing a drug. For patients with financial constraints who are able to accept close monitoring of systemic adverse effects, bevacizumab may be considered cautiously after adequate notification of the risks, while for patients who are more concerned about safety, ranibizumab may be a better choice. In addition, for patients with refractory vascular AMD and PED, alternating injections of ranibizumab and bevacizumab have been shown to improve visual acuity and reduce macular thickness in some patients, which provides a new idea for clinical treatment, but more studies are needed to validate its efficacy and safety.

Comparison of the bibliometric trends of ranibizumab with other AMD therapies (e.g., bevacizumab and abciximab) reveals that, in terms of the number of studies, the number of studies related to ranibizumab grew rapidly in the early years, but in recent years, the rate of research on abciximab increased at a faster rate. In terms of research hotspots, ranibizumab focuses on treatment regimen optimization and safety assessment; although bevacizumab is comparable to ranibizumab in terms of efficacy, more studies have focused on its safety and long-term effects due to its “over-indication” use, and abciximab, due to its long-lasting effect, has been studied more often in terms of reducing the frequency of injections and improving patient compliance. This comparison helps to provide a comprehensive picture of AMD treatments. This comparison helps to provide a comprehensive understanding of the overall trends in AMD treatment research and provides a broader perspective for subsequent studies.

Cluster 3 (Figure 6, blue cluster): The third research cluster contains 18 high-frequency keywords, such as age-related macular degeneration, optical coherence tomography (OCT), verteporfin, photodynamic therapy (PDT), polypoidal choroidal vasculopathy, etc. This part of the study focuses on the differential diagnosis between the imaging diagnosis of AMD and other fundus hemorrhagic diseases and verteporfin PDT for AMD. Clinically, most of the fundus images of AMD were identified and analyzed by fundus color photography, OCT, optical coherence tomography angiography (OCTA), and fundus angiography (FFA). Vitreous warts, intraretinal high-reflective foci (HRF) and low-reflective foci (h RF), subretinal vitreous wart-like deposits (SDD), retinal pseudophakic vitreous warts (RPD), and retinal pigment epithelium-vitreous wart complexes (RPEDCs) are imaging markers to identify and predict the progression of early- and mid-stage AMD. Vitelline wart volume is an important predictor of early or intermediate AMD progression to digraphic atrophy and MNV, and vitelline wart volume within 3 mm of the central concavity serves as a marker for identifying a high risk of progression [32]. One study [33] found HRF to be the strongest independent predictor of AMD progression and also identified four features in OCT images: (1) vitelline warts with a volume ≥ 0.03 mm^3^ within 3 mm of the center concavity, (2) presence of HRF, (3) presence of SDD, and (4) presence of h RF within the DL. A recent study [34] found that the presence of punctate SDD was independently associated with nAMD progression, with a risk three times that of the absence of punctate SDD, while the presence of fusion SDD was independently associated with geographic atrophy progression, with a risk four times that of the absence. RPEDC not only identifies intermediate AMD but is also a marker for predicting the progression of intermediate AMD. Folgar et al. [35] identified that for every 0.001 mm^3^ increase in the volume of abnormal thinning of the RPEDC at baseline, there was a 32% increase in the probability of developing a centralized geographic atrophy within 2 years.

The FFA can categorize nAMD into occult (type 1) MNV, classic (type 2) MNV, microscopic classic MNV, retinal angiomatous proliferation (RAP), and polypoid choroidal vasculopathy (PCV). Farecki et al. [36] concluded that type 1 CNVs appeared larger, were poorly demarcated from the peripheral vasculature, and were seen primarily in the choroidal capillary layer, which is in the sub-RPE layer, whereas type 2 CNVs appeared smaller, were more well demarcated, and were predominantly found in the outer layers of the retina, with more extension into the subretinal space. RAP originates from the deep retinal capillary plexus, and its initial location is within the retina, growing toward the RPE. The incidence of RAP in nAMD primary cases is 10%–15% [37, 38]. PCV was initially recognized as a choroidal disease, and the 2020 International Consensus Study Group on Nomenclature of Age-Related Macular Degeneration [39] also classified it as type 1 MNV. Frequency-domain and swept-frequency OCTs, with their high levels of specificity and sensitivity, may be an effective noninvasive alternative to indocyanine green angiography for the detection of PCV, and they may be able to effectively discriminate between PCV and cryptogenic MNV.

Verteporfin PDT was approved by the FDA for the treatment of CNV in 2000 [40]. The results of previous studies [41, 42] have confirmed that Verteporfin PDT is effective in the treatment of nAMD and that PDT is also a safer treatment. And the combination ranibizumab treatment was effective in improving BCVA and achieving higher rates of complete polyp regression with fewer ranibizumab injections [43]. However, a recent study [44] of patients with nAMD were given afliximab injections once a month for 3 months and every 2 months for the first year. After 1 year, treatment with afliximab monotherapy or in combination with PDT was used as determined by a retinal specialist. Only cases that completed 3 years of follow-up were included. The results found that vision in patients with nAMD remained at baseline levels at year 3; however, patients treated with rescue PDT developed macular atrophy more frequently and had worse vision. Currently, there is some controversy about the safety of vitepofungin PDT treatment or in combination with ranibizumab in the treatment of nAMD, and further studies are needed in the future.

Previous studies have demonstrated that PDT is effective and relatively safe in nAMD, and that combination therapy with ranibizumab is effective in improving BCVA, resulting in a higher rate of complete polyp regression and a reduction in the number of ranibizumab injections. However, recent studies have found that nAMD patients treated with remedial PDT have a higher incidence of macular atrophy and poorer visual acuity at 3 years. The safety of vitepofungin PDT alone or in combination with ranibizumab for the treatment of nAMD is currently controversial. This suggests that clinicians should weigh the efficacy and safety of vitepofungin PDT when considering its use in the treatment of nAMD and choose the treatment regimen carefully while looking forward to more studies in the future to clarify its safety and optimal treatment modality.

Cluster 4 (Figure 6, yellow cluster): The fourth research cluster contains 15 high-frequency keywords such as prevalence, eye, risk, geographic atrophy, maculopathy, visual impairment, etc. This section focuses on the epidemiology of AMD and the risk factors for the progression of macular degeneration in the fundus of the eye. Approximately 200 million people worldwide suffer from different subtypes of AMD. By 2040, the number of people with AMD is expected to increase to 288 million worldwide. In addition, the prevalence of AMD in China is increasing year by year [12]. Age is the main risk factor for the development of AMD, with smoking, increased body mass index, hypertension, hyperlipidemia, and genetics as other important risk factors [45]. Some studies [46] have shown that current smokers and those with a past history of smoking have an almost twofold increased risk of developing AMD. Adams et al. [47] suggested a modest association between alcohol consumption and an increased risk of AMD, with those who drank more than 20 mL of alcohol per day, having an approximately 20% increased risk of AMD. The intake of high-sugar diet will inevitably lead to the rise and fluctuation of blood sugar; long-term high blood sugar or blood sugar fluctuation changes too much will produce a series of damage to human organs. Rowan et al. [48] proposed that a high-sugar diet leads to characteristic AMD damage in the retina, including retinal pigment epithelial pigmentation and atrophy, lipofuscin accumulation, and photoreceptor degeneration, whereas no damage was found on a low-sugar diet. Other studies [49] have shown that smokers have a fourfold increased risk of developing AMD. Several studies [50] have shown that hypertensive patients are at an increased risk for AMD, possibly due to the effect of systolic blood pressure on choroidal blood flow. Hyperlipidemia also acts as a risk factor for AMD, with studies suggesting that HDL and high cholesterol intake may be associated with nAMD and elevated serum cholesterol, as well as the progression of macular digit-like atrophy [50]. The mice fed a high-fat diet showed a corresponding increase in oxidative stress and inflammatory markers, and the retinas of the mice exhibited pathological features of AMD [51]. Therefore, improving lifestyle and dietary habits can effectively reduce the incidence of AMD.

UV light induces significant oxidative stress in the RPE, ultimately leading to pathological changes characteristic of AMD. UV radiation inhibits RPE cell proliferation, leads to loss of transmembrane potential, and induces apoptosis in RPE cells [52]. Hypoxia has an effect on RPE metabolism, leading to degeneration of photoreceptor cells, which is a common feature of AMD [53]. In a hypoxic environment, plasma expression levels of hypoxic and inflammatory factors are increased in AMD patients, such as VEGF and erythropoietin (EPO) [54]. An in-depth study of the environmental factors affecting AMD will help to further explore the pathogenesis of AMD and new therapeutic strategies in the future.

Complement genes such as complement factor H (CFH) gene in which mutations rs1061170, rs10922109, and complement factors C2/CFB, CFI and C3 were found to be associated with the progression of AMD [55]. Serine protease (HTRA1) and age-related macular degeneration susceptibility factor 2 (ARMS2) are both found in retinal tissues, and their serum expression is significantly elevated in AMD patients [56]. AMD also has a familial susceptibility. A study by Haijes et al. compared the results of patients with AMD with those of their siblings identified through testing, and approximately 46% of those related to the AMD patient experienced the same symptoms [57]. The identification of therapeutic targets or precision methods for these susceptible or mutated genes may facilitate the diagnosis and treatment of AMD.

Mutations rs1061170 and rs10922109 in the CFH gene as well as complement factors C2/CFB, CFI, and C3 are associated with the progression of AMD, HTRA1, and ARMS2 present in retinal tissues and are significantly expressed in the sera of AMD patients, and there is a familial susceptibility to AMD. The identification of therapeutic targets or precise approaches to these susceptible or mutated genes can help in the diagnosis and treatment of AMD. Clinicians may consider genetic testing when dealing with patients with a family history of the disease for early detection and intervention to achieve precision medicine.

Cluster 5 (Figure 6, purple cluster): The fifth part of the cluster study contains 10 high-frequency keywords such as bevacizumab, aflibercept, AMD, anti-VEGF therapy, tachyphylaxis, and so on. Aflibercept is a human recombinant fusion protein that acts as a soluble VEGF family of bait receptors, including VEGF-A, VEGF-B, and PLGF, thereby inhibiting the downstream signaling mediated by these receptors. The long half-life after vitreous injection of abciximab has the advantage of reducing cost and frequency of injections. Aflibercept intravitreal solution was approved by the FDA in 2011 for the treatment of nAMD [58, 59]. There are also many clinical studies comparing the efficacy and safety of aflibercept and ranibizumab in the treatment of nAMD. Gillies et al. [60] compared the differences in macular atrophy at 24 months after the treatment of nAMD with ranibizumab and aflibercept, respectively. Patients were treated with once-monthly ranibizumab of 0.5 mg and abciximab of 2.0 mg for the first 3 months and then treated according to the T&E regimen, and the incidence and rate of progression of macular atrophy were not significantly different between the two groups at 24 months. In a clinical study with a 3-year follow-up, there was no statistically significant difference between ranibizumab and aflibercept in terms of visual acuity improvement and reduction of macular retinal thickness (CMT) in the treatment of nAMD [61]. In the absence of statistically significant differences in IOP, optic disc width, and depth, both intravitreal injections of ranibizumab and aflibercept can be considered safe options for patients with nAMD combined with primary open-angle glaucoma (POAG) [62].

Clinically, when performing an anti-VEGF drug is ineffective or unresponsive, we consider switching drugs. Queguiner et al. [63] retrospectively analyzed 33 cases of 38 eyes of AMD patients with suboptimal response to treatment with ranibizumab or relapsed AMD who were switched to aflibercept. The results showed no statistically significant difference in visual acuity improvement or macular retinal anatomy improvement regardless of the reason for the “switch,” but among the “suboptimal” patients, those who switched from ranibizumab to aflibercept had significantly fewer follow-up visits and fewer injections. In “suboptimal” patients, however, switching from rezumab to aflibercept significantly reduced the number of follow-up visits and injections. In addition, the clinical use of aflibercept and ranibizumab in the treatment of nAMD was safe, and the adverse effects that occurred could be treated and recovered within a short period of time, and no serious adverse effects occurred.

In addition, abciximab and ranibizumab are safe for clinical use in the treatment of nAMD, and the adverse reactions can be recovered within a short period of time, with no serious adverse reactions occurring. This suggests that abciximab is an option for clinicians when choosing anti-VEGF drugs for nAMD, taking into account the cost and frequency of injections, and that switching to abciximab is a feasible strategy when the effect of ranibizumab treatment is poor, and the safety of switching to abciximab is guaranteed.

Based on the bibliometric data, in addition to the mentioned research hotspots, the emerging trend is also reflected in the exploration of the combined application of gene therapy and ranibizumab, and the related research may bring new breakthroughs in the treatment of AMD. In terms of research gaps, the long-term effects of ranibizumab in the treatment of AMD have not yet been adequately assessed, especially the long-term effects on patients' quality of life and potential long-term complications. In addition, differences in response to treatment with ranibizumab in different ethnic and geographic populations need to be studied in depth. For researchers, these bibliometric data can be utilized to further clarify research directions. For example, basic researchers can focus on mining new therapeutic targets and combining gene editing technology with ranibizumab therapy; clinicians can conduct large-scale, multicenter long-term follow-up studies to assess the differences in therapeutic efficacy among different populations and provide a basis for personalized treatment.

## 5. Conclusion

To the best of our knowledge, the present study offers the first comprehensive bibliometric analysis of research pertaining to ranibizumab and AMD. By examining publications over the past nearly 15 years, this bibliometric visualization delves into the dynamic evolution of research on ranibizumab and AMD. It encompasses aspects such as publication trends, global collaborative patterns, and research hotspots. The insights gleaned from these findings empower the research community to discern emerging topics and forefront research directions, thereby providing guidance for future studies. This analysis underscores the critical role of sustainable anti-VEGF strategies in AMD management. Future research should prioritize (1) real-world cost–benefit comparisons of approved therapies, (2) environmental impact assessments of intravitreal drug delivery systems, and (3) equitable access models for low-resource settings.

## Figures and Tables

**Table 1:** The top 10 journals for ranibizumab and AMD publications.

Rank	Source	IF(2023)/JCR	Total publications (Percentage)	Total citations
1	RETINA THE JOURNAL OF RETINAL AND VITREOUS DISEASES	3.3/Q2	285 (10.591%)	6496
2	OPHTHALMOLOGY	13.7/Q1	193 (7.172%)	20,865
3	GRAEFES ARCHIVE FOR CLINICAL AND EXPERIMENTAL OPHTHALMOLOGY	2.7/Q2	163 (6.057%)	2540
4	AMERICAN JOURNAL OF OPHTHALMOLOGY	4.2/Q1	120 (4.459%)	5226
5	BRITISH JOURNAL OF OPHTHALMOLOGY	4.1/Q1	120 (4.459%)	4136
6	EYE	3.9/Q1	95 (3.530%)	2369
7	ACTA OPHTHALMOLOGICA	3.4/Q2	89 (3.307%)	1533
8	OPHTHALMOLOGICA	2.6/Q2	89 (3.307%)	1072
9	INVESTIGATIVE OPHTHALMOLOGY VISUAL SCIENCE	4.4/Q1	78 (2.899%)	2731
10	EUROPEAN JOURNAL OF OPHTHALMOLOGY	1.7/Q4	66 (2.453%)	354

**Table 2:** The top 10 highest cited articles.

Title	Journal	Citations	Avg citations	PY
Global prevalence of age-related macular degeneration and disease burden projection for 2020 and 2040: A systematic review and meta-analysis [12]	LANCET GLOB HEALTH	2561	256.1	2014
Ranibizumab and bevacizumab for neovascular age-related macular degeneration of the CATT research group [13]	NEW ENGLAND JOURNAL OF MEDICINE	2010	154.62	2011
Intravitreal aflibercept (VEGF trap-eye) in wet age-related macular degeneration [14]	OPHTHALMOLOGY	1602	133.5	2012
Ranibizumab and bevacizumab for the treatment of neovascular age-related macular degeneration [15]	OPHTHALMOLOGY	1336	111.3	2012
Age-related macular degeneration [16]	LANCET	1225	104.58	2012
The restore study ranibizumab monotherapy or combined with laser versus laser monotherapy for diabetic macular edema [17]	OPHTHALMOLOGY	1000	76.92	2011
Autologous induced stem-cell-derived retinal cells for macular degeneration [18]	NEW ENGLAND JOURNAL OF MEDICINE	857	122.43	2017
Seven-year outcomes in ranibizumab-treated patients in anchor, marina, and horizon [19]	OPHTHALMOLOGY	716	65.09	2013
Binding and neutralization of vascular endothelial growth factor (VEGF) and related ligands by VEGF trap, ranibizumab, and bevacizumab [20]	ANGIOGENESIS	713	59.42	2012
Ranibizumab versus bevacizumab to treat neovascular age-related macular degeneration [21]	OPHTHALMOLOGY	612	51	2012

**Table 3:** The top 10 productive countries/regions.

Rank	Country/region	Publications	Citations
1	USA	786	38,014
2	Germany	319	12,583
3	England	277	10,862
4	Switzerland	254	7720
5	Japan	250	7870
6	Peoples R China	219	3708
7	South Korea	199	2795
8	France	183	7870
9	Australia	182	7407
10	Italy	169	5331

**Table 4:** The top 10 productive institutions.

Rank	Institution	Country	Publications	Citations
1	University of Sydney	Australia	98	5245
2	University of California genentech	USA	73	3836
3	University of Melbourne	Australia	67	3018
4	Medical University of Vienna	Germany	66	6510
5	Cleveland Clinic	USA	59	6546
6	Johns Hopkins University	USA	59	4413
7	University of Konyang	South Korea	55	597
8	University of Zurich	Switzerland	54	2335
9	University of Pennsylvania	USA	53	7064
10	University of Bonn	Germany	50	4190

**Table 5:** The top 10 productive authors.

Rank	Author	Publications	Citations
1	Kim, Jong Woo	56	550
2	Schmidt-Erfurth, Ursula	48	5890
3	Kim, Chul Gu	48	424
4	Barthelmes, Daniel	45	1372
5	Kim, Jae Hui	45	427
6	Souied, Eric H.	40	1005
7	Maguire, Maureen G.	39	6535
8	Holz, Frank G.	39	3960
9	Jaffe, Glenn J.	37	7469
10	Gillies, Mark C.	37	6369

**Table 6:** The top 10 keywords of ranibizumab and AMD.

Rank	Keywords	Occurrence
1	Ranibizumab	1919
2	Age-related macular degeneration	831
3	Bevacizumab	812
4	Macular degeneration	665
5	Choroidal neovascularization	521
6	Therapy	474
7	Aflibercept	457
8	Endothelial growth factor	445
9	Optical coherence tomography	350
10	Verteporfin	300

**Table 7:** Clusters of the top 100 keywords.

Cluster	Keywords	Counts
1	Therapy	474
1	Outcomes	299
1	Neovascular age-related macula	241
1	Safety	236
1	Visual acuity	222
1	Efficacy	219
1	Intravitreal ranibizumab	208
1	Trial	138
1	Anti-vascular endothelial grow	127
1	Subgroup analysis	91
1	Dosing regimen	87
1	Visual acuity	81
1	Regimen	73
1	Ranibizumab treatment	71
1	Anchor	69
1	Subfoveal choroidal neovascular	68
1	Growth-factor therapy	65
1	2.0 mg ranibizumab	62
1	Intravitreal injections	61
1	Treat-and-extend	56
1	Neovascular AMD	50
1	Quality of life	49
1	Marina	48
1	Predictors	48
1	Atrophy	44
1	Morphology	44
1	Follow-up	43
1	Vision	42
1	Acuity	42
1	Exudative age-related macular	40
2	Ranibizumab	1919
2	Macular degeneration	665
2	Choroidal neovascularization	521
2	Endothelial growth factor	445
2	Anti-VEGF	234
2	VEGF	227
2	Intravitreal injections	166
2	Intravitreal bevacizumab	134
2	Angiogenesis	121
2	Retina	113
2	Expression	101
2	Vascular endothelial growth factor	109
2	Degeneration	90
2	Pharmacokinetics	86
2	Diabetic macular edema	74
2	Edema	72
2	Avastin	70
2	Cells	62
2	Pegaptanib	57
2	Injections	53
2	Inflammation	52
2	Endophthalmitis	49
2	Retina-pigment epithelium	44
2	Ranibizumab	43
2	Retina vein occlusion	40
2	Macula	40
2	Factor therapy	34
3	Age-related macular degeneration	831
3	Optical coherence tomography	350
3	Verteporfin	300
3	Photodynamic therapy	273
3	Injection	195
3	Polypoidal choroidal vasculopathy	173
3	Secondary	98
3	Neovascularization	94
3	Verteporfin photodynamic therapy	88
3	Management	86
3	Pigment epithelial detachment	74
3	Retinal angiomatous proliferation	70
3	Natural history	56
3	Thickness	53
3	Subretinal hemorrhage	47
3	Occult choroidal neovascularization	38
3	Fluorescein angiography	37
3	Indocyanine green angiography	33
4	Prevalence	232
4	Eye	154
4	Risk	145
4	Geographic atrophy	132
4	Disease	108
4	Maculopathy	105
4	Visual impairment	87
4	Association	86
4	Progression	84
4	Risk factors	75
4	Blindness	58
4	Complement factor-h	54
4	Growth	51
4	Choroidal thickness	44
4	Population	41
5	Bevacizumab	812
5	Aflibercept	457
5	Eyes	172
5	AMD	166
5	Intravitreal aflibercept	137
5	Anti-VEGF therapy	107
5	Tachyphylaxis	73
5	VEGF trap	63
5	VEGF-trap	60
5	Fluid	45

## Data Availability

The raw data supporting the conclusions of this article will be made available by the authors, without undue reservation. Raw data packages needed for this study can be obtained by contacting the corresponding author and the first author of this paper. Complete disclosure forms are available upon request.

## References

[1] Jaffe G., Westby K., Csaky K. (2021). C5 Inhibitor Avacincaptad Pegol for Geographic Atrophy Due to Age-Related Macular Degeneration: A Randomized Pivotal Phase 2/3 Trial. *Ophthalmology*.

[2] Kaarniranta K., Blasiak J., Liton P., Boulton M., Klionsky D., Sinha D. (2023). Autophagy in Age-Related Macular Degeneration. *Autophagy*.

[3] Kaarniranta K., Uusitalo H., Blasiak J. (2020). Mechanisms of Mitochondrial Dysfunction and Their Impact on Age-Related Macular Degeneration. *Progress in Retinal and Eye Research*.

[4] Dugel P., Singh R., Koh A. (2021). HAWK and HARRIER: Ninety-Six-Week Outcomes from the Phase 3 Trials of Brolucizumab for Neovascular Age-Related Macular Degeneration. *Ophthalmology*.

[5] Told R., Reiter G., Mittermüller T. J. (2021). Profiling Neovascular Age-Related Macular Degeneration Choroidal Neovascularization Lesion Response to Anti-vascular Endothelial Growth Factor Therapy Using SSOCTA. *Acta Ophthalmologica*.

[6] Ferro Desideri L., Barra F., Ferrero S., Traverso C., Nicolò M. (2019). Clinical Efficacy and Safety of Ranibizumab in the Treatment of Wet Age-Related Macular Degeneration. *Expert Opinion on Biological Therapy*.

[7] Agarwal A., Durairajanayagam D., Tatagari S. (2016). Bibliometrics: Tracking Research Impact by Selecting the Appropriate Metrics. *Asian Journal of Andrology*.

[8] Peng C., Kuang L., Zhao J., Ross A., Wang Z., Ciolino J. (2022). Bibliometric and Visualized Analysis of Ocular Drug Delivery from 2001 to 2020. *Journal of Controlled Release*.

[9] van Eck N., Waltman L. (2010). Software Survey: VOSviewer, a Computer Program for Bibliometric Mapping. *Scientometrics*.

[10] Newman M. (2004). Coauthorship Networks and Patterns of Scientific Collaboration. *Proceedings of the National Academy of Sciences*.

[11] Li J., Yin Y., Fortunato S., Wang D. (2020). Scientific Elite Revisited: Patterns of Productivity, Collaboration, Authorship and Impact. *Journal of the Royal Society, Interface*.

[12] Wong W., Su X., Li X. (2014). Global Prevalence of Age-Related Macular Degeneration and Disease Burden Projection for 2020 and 2040: a Systematic Review and Meta-Analysis. *Lancet Global Health*.

[13] Martin D. F., Maguire M. G., Ying G., Grunwald J. E., Fine S. L., Jaffe G. J. (2011). Ranibizumab and Bevacizumab for Neovascular Age-Related Macular Degeneration. *New England Journal of Medicine*.

[14] Heier J. S., Brown D. M., Chong V. (2012). Intravitreal Aflibercept (VEGF Trap-Eye) in Wet Age-Related Macular Degeneration. *Ophthalmology*.

[15] Martin D., Maguire M., Fine S. (2012). Ranibizumab and Bevacizumab for Treatment of Neovascular Age-Related Macular Degeneration: Two-Year Results. *Ophthalmology*.

[16] Lim L., Mitchell P., Seddon J., Holz F., Wong T. (2012). Age-related Macular Degeneration. *The Lancet*.

[17] Mitchell P., Bandello F., Schmidt-Erfurth U. (2011). The RESTORE Study: Ranibizumab Monotherapy or Combined with Laser versus Laser Monotherapy for Diabetic Macular Edema. *Ophthalmology*.

[18] Mandai M., Watanabe A., Kurimoto Y. (2017). Autologous Induced Stem-Cell-Derived Retinal Cells for Macular Degeneration. *New England Journal of Medicine*.

[19] Rofagha S., Bhisitkul R., Boyer D., Sadda S., Zhang K. (2013). Seven-year Outcomes in Ranibizumab-Treated Patients in ANCHOR, MARINA, and HORIZON: a Multicenter Cohort Study (SEVEN-UP). *Ophthalmology*.

[20] Papadopoulos N., Martin J., Ruan Q. (2012). Binding and Neutralization of Vascular Endothelial Growth Factor (VEGF) and Related Ligands by VEGF Trap, Ranibizumab and Bevacizumab. *Angiogenesis*.

[21] Chakravarthy U., Harding S., Rogers C. (2012). Ranibizumab versus Bevacizumab to Treat Neovascular Age-Related Macular Degeneration: One-Year Findings from the IVAN Randomized Trial. *Ophthalmology*.

[22] Kim L., Mehta H., Barthelmes D., Nguyen V., Gillies M. (2016). METAANALYSIS OF REAL-WORLD OUTCOMES OF INTRAVITREAL RANIBIZUMAB FOR THE TREATMENT OF NEOVASCULAR AGE-RELATED MACULAR DEGENERATION. *Retina*.

[23] Silva R., Berta A., Larsen M., Macfadden W., Feller C., Monés J. (2018). Treat-and-Extend versus Monthly Regimen in Neovascular Age-Related Macular Degeneration: Results with Ranibizumab from the TREND Study. *Ophthalmology*.

[24] Ho A., Busbee B., Regillo C. (2014). Twenty-four-month Efficacy and Safety of 0.5 Mg or 2.0 Mg Ranibizumab in Patients with Subfoveal Neovascular Age-Related Macular Degeneration. *Ophthalmology*.

[25] Matsuda S., Gomi F., Oshima Y., Tohyama M., Tano Y. (2005). Vascular Endothelial Growth Factor Reduced and Connective Tissue Growth Factor Induced by Triamcinolone in ARPE19 Cells under Oxidative Stress. *Investigative Opthalmology & Visual Science*.

[26] Lowe J., Araujo J., Yang J. (2007). Ranibizumab Inhibits Multiple Forms of Biologically Active Vascular Endothelial Growth Factor In Vitro and In Vivo. *Experimental Eye Research*.

[27] Matts K., Oskar A., Klas P. (2023). Maass: Population Pharmacokinetics of Ranibizumab Delivered via the Port Delivery System Implanted in the Eye in Patients With Neovascular Age-Related Macular Degeneration. *The Journal of Clinical Pharmacology*.

[28] Bressler N., Kim T., Oh I., Russo P., Kim M., Woo S. (2023). Immunogenicity with Ranibizumab Biosimilar SB11 (Byooviz) and Reference Product Lucentis and Association with Efficacy, Safety, and Pharmacokinetics: A Post Hoc Analysis of a Phase 3 Randomized Clinical Trial. *JAMA ophthalmology*.

[29] Fong D., Custis P., Howes J., Hsu J. (2010). Intravitreal Bevacizumab and Ranibizumab for Age-Related Macular Degeneration. *Ophthalmology*.

[30] Witkin A., Rayess N., Garg S. (2017). Alternating Bi-weekly Intravitreal Ranibizumab and Bevacizumab for Refractory Neovascular Age-Related Macular Degeneration with Pigment Epithelial Detachment. *Seminars in Ophthalmology*.

[31] Avery R. L., Castellarin A. A., Steinle N. C. (2017). Hanley: systemic Pharmacokinetics and Pharmacodynamics of Intravitreal Aflibercept, Bevacizumab, and Ranibizumab. *Retina*.

[32] Abdelfattah N., Zhang H., Boyer D. (2016). Drusen Volume as a Predictor of Disease Progression in Patients with Late Age-Related Macular Degeneration in the Fellow Eye. *Investigative Opthalmology & Visual Science*.

[33] Lei J., Balasubramanian S., Abdelfattah N., Nittala M., Sadda S. (2017). Proposal of a Simple Optical Coherence Tomography-Based Scoring System for Progression of Age-Related Macular Degeneration. *Graefes Archive for Clinical and Experimental Ophthalmology*.

[34] Zhou Q., Daniel E., Maguire M. (2016). Pseudodrusen and Incidence of Late Age-Related Macular Degeneration in Fellow Eyes in the Comparison of Age-Related Macular Degeneration Treatments Trials. *Ophthalmology*.

[35] Folgar F., Yuan E., Sevilla M. (2016). Drusen Volume and Retinal Pigment Epithelium Abnormal Thinning Volume Predict 2-Year Progression of Age-Related Macular Degeneration. *Ophthalmology*.

[36] Farecki M., Gutfleisch M., Faatz H. (2017). Characteristics of Type 1 and 2 CNV in Exudative AMD in OCT-Angiography. *Graefes Archive for Clinical and Experimental Ophthalmology*.

[37] Sasaki M., Kawasaki R., Yanagi Y. (2022). Early Stages of Age-Related Macular Degeneration: Racial/Ethnic Differences and Proposal of a New Classification Incorporating Emerging Concept of Choroidal Pathology. *Journal of Clinical Medicine*.

[38] Faatz H., Rothaus K., Ziegler M. (2022). Vascular Analysis of Type 1, 2, and 3 Macular Neovascularization in Age-Related Macular Degeneration Using Swept-Source Optical Coherence Tomography Angiography Shows New Insights into Differences of Pathologic Vasculature and May Lead to a More Personalized Understanding. *Biomedicines*.

[39] Spaide R. F. (2020). Consensus Nomenclature for Reporting Neovascular Age-Related Macular Degeneration Data: Consensus on Neovascular Age-Related Macular Degeneration Nomenclature Study Group. *Ophthalmology*.

[40] Pitlick J., Vecera K., Barnes K., Reski J., Forinash A. (2012). Bevacizumab for the Treatment of Neovascular Age-Related Macular Degeneration. *The Annals of Pharmacotherapy*.

[41] Hatz K., Schneider U., Henrich P., Braun B., Sacu S., Prünte C. (2015). Ranibizumab Plus Verteporfin Photodynamic Therapy in Neovascular Age-Related Macular Degeneration: 12 Months of Retreatment and Vision Outcomes from a Randomized Study. *Ophthalmologica*.

[42] Kaiser P., Boyer D., Cruess A., Slakter J., Pilz S., Weisberger A. (2012). Verteporfin Plus Ranibizumab for Choroidal Neovascularization in Age-Related Macular Degeneration: Twelve-Month Results of the DENALI Study. *Ophthalmology*.

[43] Takahashi K., Ohji M., Terasaki H. (2018). Efficacy and Safety of Ranibizumab Monotherapy versus Ranibizumab in Combination with Verteporfin Photodynamic Therapy in Patients with Polypoidal Choroidal Vasculopathy: 12-month Outcomes in the Japanese Cohort of EVEREST II Study. *Clinical Ophthalmology*.

[44] Yoshida M., Oishi A., Miyake M. (2022). Rescue Photodynamic Therapy for Age-Related Macular Degeneration Refractory to Anti-vascular Endothelial Growth Factor Monotherapy. *Photodiagnosis and Photodynamic Therapy*.

[45] Casten R., Rovner B. (2013). Update on Depression and Age-Related Macular Degeneration. *Current Opinion in Ophthalmology*.

[46] Seddon J., George S., Rosner B. (2006). Cigarette Smoking, Fish Consumption, Omega-3 Fatty Acid Intake, and Associations with Age-Related Macular Degeneration: the US Twin Study of Age-Related Macular Degeneration. *Archives of Ophthalmology*.

[47] Adams M. K. M., Chong E. W., Williamson E. (2012). 20/20--Alcohol and Age-Related Macular Degeneration: The Melbourne Collaborative Cohort Study. *American Journal of Epidemiology*.

[48] Rowan S., Jiang S., Korem T. (2017). Involvement of a Gut-Retina axis in Protection against Dietary Glycemia-Induced Age-Related Macular Degeneration. *Proceedings of the National Academy of Sciences*.

[49] Joachim N., Mitchell P., Burlutsky G., Kifley A., Wang J. (2015). The Incidence and Progression of Age-Related Macular Degeneration over 15 years: The Blue Mountains Eye Study. *Ophthalmology*.

[50] Pennington K., DeAngelis M. (2016). Epidemiology of Age-Related Macular Degeneration (AMD): Associations with Cardiovascular Disease Phenotypes and Lipid Factors. *Eye and vision (London, England)*.

[51] Tuzcu M., Orhan C., Muz O., Sahin N., Juturu V., Sahin K. (2017). Lutein and Zeaxanthin Isomers Modulates Lipid Metabolism and the Inflammatory State of Retina in Obesity-Induced High-Fat Diet Rodent Model. *BMC Ophthalmology*.

[52] Schick T., Ersoy L., Lechanteur Y. (2016). HISTORY OF SUNLIGHT EXPOSURE IS A RISK FACTOR FOR AGE-RELATED MACULAR DEGENERATION. *Retina*.

[53] Kurihara T., Westenskow P. D., Gantner M. L. (2016). Hypoxia-induced Metabolic Stress in Retinal Pigment Epithelial Cells Is Sufficient to Induce Photoreceptor Degeneration. *Elife*.

[54] Ioanna Z., Christian S., Christian G., Daniel B. (2018). Plasma Levels of Hypoxia-Regulated Factors in Patients with Age-Related Macular Degeneration. *Graefes Archive for Clinical and Experimental Ophthalmology*.

[55] Yan Q., Ding Y., Liu Y. (2018). Genome-wide Analysis of Disease Progression in Age-Related Macular Degeneration. *Human Molecular Genetics*.

[56] Qureshi I., Ambreen F. (2017). Serum APOE, Leptin, CFH and HTRA1 Levels in Pakistani Age Related Macular Degeneration Patients. *JPMA The Journal of the Pakistan Medical Association*.

[57] Haijes H. A., Molema F., Langeveld M. (2020). Retrospective Evaluation of the Dutch Pre-newborn Screening Cohort for Propionic Acidemia and Isolated Methylmalonic Acidemia: What to Aim, Expect, and Evaluate from Newborn Screening?. *Journal of Inherited Metabolic Disease*.

[58] Ba J., Peng R. S., Xu D. (2015). Intravitreal Anti-VEGF Injections for Treating Wet Age-Related Macular Degeneration: a Systematic Review and Meta-Analysis. *Drug Design, Development and Therapy*.

[59] Schmucker C., Rücker G., Sommer H. (2015). Treatment as Required versus Regular Monthly Treatment in the Management of Neovascular Age-Related Macular Degeneration: A Systematic Review and Meta-Analysis. *PLoS One*.

[60] Gillies M., Hunyor A., Arnold J. (2020). Macular Atrophy in Neovascular Age-Related Macular Degeneration: A Randomized Clinical Trial Comparing Ranibizumab and Aflibercept (RIVAL Study). *Ophthalmology*.

[61] Bhandari S., Nguyen V., Arnold J. (2020). Treatment Outcomes of Ranibizumab versus Aflibercept for Neovascular Age-Related Macular Degeneration: Data from the Fight Retinal Blindness! Registry. *Ophthalmology*.

[62] Rud’ko A., Budzinskaya M., Andreeva I., Karpilova M., Smirnova T. (2019). [Effect of Intravitreal Injections of Ranibizumab and Aflibercept on the Retinal Nerve Fiber Layer in Patients with Concomitant Neovascular Age-Related Macular Degeneration and Glaucoma]. *Vestnik Oftalmologii*.

[63] Queguiner F., Bezirganyan K., Courjaret J., Curel L., Penaranda G., Righini Chossegros M. (2020). Impact of Switching from Ranibizumab to Aflibercept on the Number of Intravitreous Injection and Follow up Visit in Wet AMD: Results of Real Life ELU Study. *International Journal of Ophthalmology*.

[64] Xiaodong L., Xuewei Q., Dandan W., Yi Y., Zhilin L. (2023). Ranibizumab for the Treatment of Age-Related Macular Degeneration Related Research Hotspot and Dynamic.

